# Whole-body inhalation study of nanoparticle-enhanced vegetable oil metalworking fluids in mice for assessing occupational health risks

**DOI:** 10.1038/s41598-025-06266-5

**Published:** 2025-07-01

**Authors:** Muralidhar Vardhanapu, Phaneendra Kiran Chaganti, Aparajita Ghosh, Ashutosh Mahale, Onkar Prakash Kulkarni

**Affiliations:** 1 Department of Mechanical Engineering, Birla Institute of Technology and Science-Pilani, Hyderabad campus, Jawahar Nagar, Hyderabad, 500078 India; 2 Department of Pharmacy, Birla Institute of Technology and Science-Pilani, Hyderabad campus, Jawahar Nagar, Hyderabad, 500078 India

**Keywords:** Nanoparticles, Metalworking fluids, Occupational safety, Whole-body inhalation, Sustainable cutting fluids, Cancer epidemiology, Lung cancer, Risk factors, Nanotoxicology, Mechanical engineering, Sustainability

## Abstract

The increasing demand for sustainable machining necessitates the development and toxicological evaluation of environmentally friendly alternatives to conventional metalworking fluids (MWFs). This study investigated the respiratory and systemic effects of a nanoparticle-enhanced vegetable oil-based MWF (NPVO-MWF) using a whole-body inhalation exposure model in male C57BL/6 mice. Both sub-acute (4-week) and sub-chronic (13-week) exposures were performed, followed by detailed bioanalytical and histopathological assessments. Cytokine analyses (IL-1β, TNF-α, IL-6) via ELISA and RT-PCR revealed no significant pro-inflammatory responses. Reactive oxygen species (ROS) levels in blood, bronchoalveolar lavage fluid, and lung homogenates remained unchanged, indicating an absence of oxidative stress. Histopathological analysis using hematoxylin and eosin staining showed mild epithelial hyperplasia in sub-chronic exposures. Sirius Red staining revealed minimal collagen deposition and slight reductions in pulmonary airspace. No substantial evidence of lung injury, pulmonary toxicity, or carcinogenicity was observed. This study represents one of the first applications of a whole-body inhalation model for evaluating NPVO-MWFs, addressing a critical gap in occupational health risk assessment. The findings indicate that NPVO-MWFs present low toxicological risk under the tested conditions and offer a viable, sustainable alternative to conventional MWFs. Additional chronic exposure studies are recommended to further establish their occupational safety.

## Introduction

Metalworking fluids (MWFs), often termed cutting fluids (CFs), are available in various types including petroleum-based, natural (plant or vegetable-based), and synthetic or semi-synthetic formulations. These fluids play a vital role in machining operations by enhancing lubrication, providing cooling, improving tool performance, and removing swarf. Their widespread use is evident in industries such as automotive, aerospace, and general manufacturing, where precise metal shaping and processing are critical. Compared with dry machining—commonly known as green processing technology—MWFs (wet machining) significantly improve performance in terms of surface finish, dimensional accuracy, tool life, and overall manufacturing efficiency^1^. However, MWFs present significant challenges, including their alarmingly increasing global usage, the incorporation of chemicals and additives of unknown toxicity, and their overall potential to pose environmental and health hazards.

As alternatives to traditional mineral oil-based (petroleum-derived) cutting fluids, which contain toxic polyaromatic and naphthenic hydrocarbons, the development and use of synthetic and vegetable oil-based MWFs have increased over the past decade to address environmental and health concerns^2,3^. These alternative formulations offer benefits such as foam reduction and extended shelf-life, yet they have not fully addressed the persistent concerns related to toxicity^4,5^. While straight vegetable oil-MWFs are highly biodegradable, they often struggle to provide sufficient lubrication for heavy-duty operations and multi-metal machining^6^. Additionally, advanced synthetic fluids are often linked to the use of chemicals with unknown toxicological profiles and entail high production costs^7^. To address the lubricity and thermo-oxidative stability challenges of straight vegetable oil-MWFs, researchers have incorporated performance-enhancing additives—including lubricating agents, friction modifiers, ionic liquids, extreme-pressure additives, and nanoparticles (NPs) as emulsifiers—into base oils^8,9^. During the second decade of the 21st century, research focused on using NPs to improve the lubricity of straight vegetable oils, demonstrating that NP-modified vegetable oil-MWFs are among the most effective formulations for addressing these challenges^10–13^. In parallel, the advent of advanced MWF delivery technologies based on aerosolization principles has significantly reduced excessive fluid usage in machining. Techniques such as Minimum Quantity Lubrication (MQL) and hybrid systems—including Nanofluid-MQL, Cryo-MQL, and Ranque-Hilsch Vortex Tube MQL—have emerged as transformative solutions. These state-of-the-art methods optimize fluid consumption while enhancing machining performance by precisely targeting the cutting zone. By integrating innovative approaches, these systems reduce waste and promote sustainability, aligning with the growing demand for eco-friendly and efficient manufacturing practices^14–23^. These near-to-dry advanced MWF-delivery technologies, when combined with novel NP-enhanced vegetable oil-MWF (NPVO-MWF) formulations, may have significantly reduced MWF consumption while addressing toxicity and tribological challenges^24,25^. Despite these technological advancements, significant concerns remain, particularly regarding the health hazards associated with MWF mists. MWF mist can be generated mechanically during machining operations, through fluid delivery (sprays), or by fluid-atmosphere interactions near the cutting zone (e.g., condensation of vapours)^26^. Issues such as workers’ exposure to hazardous MWF mist and fumes, fluid disposal, and potential ecological and eco-toxicological impacts remain notable challenges for these MWF systems. Among these concerns, occupational exposure to MWF mist is a particularly debated topic within the research and development (R&D) community, the lubricating oil industry, and regulatory bodies such as the National Institute for Occupational Safety and Health (NIOSH), the Occupational Safety and Health Administration (OSHA), and the United States Environmental Protection Agency (USEPA). MWFs can enter the human body through various pathways, posing risks to operators who are often exposed to these substances for prolonged periods during their careers. From this perspective, it is critically important to evaluate the potential health impacts of novel MWF formulations, particularly given the prolonged and repeated exposure faced by automotive machinists.

The Centers for Disease Control and Prevention (CDC), in collaboration with the NIOSH, reported in their official blog dated October 6, 2008, that over 100 million gallons of MWFs are produced annually, exposing more than one million U.S. employees^27^. Worldwide, exposure to MWFs is believed to affect millions of workers, with current exposure levels potentially approaching critical thresholds. From the 1990s to the early 21st century, numerous epidemiological studies have examined the associations between exposure to different types of MWFs and the incidence of cancer, respiratory disorders, and skin conditions in occupational settings^28^. Several cohort studies conducted by researchers in collaboration with NIOSH, involving populations of over 35,000 autoworkers and, in some cases, even larger groups, found an elevated risk of esophageal, laryngeal, and lung cancers, with standardized mortality ratios (SMRs) ranging from 1.2 to 1.7 depending on the duration and type of MWF exposure^29–36^. Furthermore, these studies revealed that workers exposed to straight-oil MWFs had an approximately 70% greater risk of developing respiratory illnesses, while those exposed to water-based MWFs had a 30% higher risk of lung cancer compared to control group workers. Related studies have also shown that the risk of chronic bronchitis and work-related asthma (WRA) is increased 2.4-fold among autoworkers exposed specifically to straight oil-based MWFs^37–39^. Additionally, exposure to these MWFs has been linked to dermatological issues, including allergic contact dermatitis^40^. Building on these findings, a longitudinal study revealed that workers exposed to synthetic and semi-synthetic metalworking fluids (MWFs) experienced a significant decline in forced expiratory volume (FEV1) over a 10-year period compared to non-exposed workers^41^. This decline in lung function indicates a chronic impact resulting from prolonged inhalation of MWF mist. Several researchers have conducted cohort, case-control, and cross-sectional studies examining MWF exposure and its impact on workers in various manufacturing industries^36,42–55^. Table 1 summarizes the health conditions diagnosed among machinists, as reported in these studies and cohort analyses conducted over the last two decades.

**Table 1:** Summary of health risks identified in various studies over the last two decades.

Year	Authors	Type of study	Health risk	Type of MWF-exposure
2001	Eisen, E.A. et al.^38^	Cohort Study	1. Associated with esophageal, laryngeal and rectal cancers 2. Associated with esophageal, larynx, skin, and brain cancers 3. Associated with esophageal, liver, and prostate cancers	1. Straight MWFs 2. Soluble MWFs 3. Synthetic MWFs
2002	Thurston, S.W. et al.^37^	Cohort Study	Increased risk for prostate cancer with culminative exposure	Soluble MWFs
2003	Mirer, F.^50^	Review	Elevated risk ratios for stomach cancer	Petroleum-based MWFs
2003	Savitz, D. A.^54^	Review	Elevated risk of rectal and laryngeal cancer	Straight MWFs
2005	Agalliu, I. et al.^46^	Nested case-control study	Increased risk of prostate cancer	Soluble and straight MWFs
2010	Lillienberg, L. et al.^47^	Cross-sectional study	Increased upper and lower respiratory symptoms	Synthetic-MWF aerosol
2011	Costello, S.^49^	Cohort study	Incidence of malignant melanoma	Straight (mineral-oil based) MWFs
2011	Melissa, C. F.^48^	Cohort study	High risk of colon cancer in female autoworkers	Straight MWFs
2011	Joanne S. C. et al.^45^	Case-control study	Elevated risk of bladder cancer	Straight MWFs
2014	C. M. Barber et al.^51^	Case-control study	Hypersensitivity pneumonitis	Straight MWF
2014	P. Sherwood Burge^55^	Review	Hypersensitivity pneumonitis	MWF aerosol
2014	Colt JS et al.^56^	Nested case-control study	1. Risk of bladder cancer increased steadily with the cumulative exposure 2. 50% Increased risk of bladder cancer	1. Straight MWFs 2. Soluble MWFs
2016	Shrestha, D. et al.^52^	Cohort study	Increased risk of renal cell carcinoma	Straight MWFs
2018	Park, R. M.^53^	Risk assessment	Substantial cancer risk of larynx, esophagus, brain, female breast, and uterine cervix	Soluble oils
2018	Erika, G. et al.^57^	Cohort study	Slightly elevated risk of lung cancer mortality	Synthetic MWFs
2019	Monika, A. I.^58^	Cohort study	Incidence of colon cancer	Straight MWFs
2022	H. L. Colbeth et al.^36^	Cohort study	1. Increased incidence of stomach and kidney cancer 2. Increased rectal and prostate cancer	1. Straight MWFs 2. Water-soluble MWFs

Straight and soluble oil MWFs have a long history, spanning over a century, during which they have been subjected to extensive environmental and health risk assessments. These assessments include a wide range of studies—epidemiological, cohort, cross-sectional, nested case-control, and analytical—that have examined issues such as aerosol generation and its impact on worker safety. These efforts have been further reinforced by investigations into occupational outbreaks within prominent manufacturing industries in the United States (USA) and the United Kingdom (UK), along with regulatory oversight from agencies such as NIOSH and OSHA, resulting in comprehensive occupational and environmental characterizations^59,60^. In contrast, such in-depth research, particularly on aerosol-related risks, is significantly lacking in the current literature concerning nanoparticle-enhanced vegetable oil-MWFs (NPVO-MWFs), which have emerged in recent years. Furthermore, methodologies for assessing the potential adverse effects of these formulations, predominantly in the form of MQL mist, on machining operators’ health prior to their market introduction are notably scarce.

The authors, in their previous work, conducted several ecological and eco-toxicological studies, including in-vitro analyses using the MTT assay on WI-38 normal human lung fibroblast cells. These studies demonstrated that the developed NPVO-MWFs did not induce sudden cell death or significant changes in cell viability upon direct exposure to the cells^61^. A similar comparative study on soybean-neem oil cutting fluids formulated with anhydrous powders of gum acacia and guar gum demonstrated 100% cell viability in cytocompatibility assays, outperforming commercial-grade metalworking fluids that exhibited only 60% cell viability^62^. Although these studies suggest that NPVO-MWFs are not cytotoxic, evaluating their health risks in dynamic machining environments—where MWF aerosols and various factors interact—remains essential. The whole-body inhalation procedure using animal models, such as guinea pigs, rabbits, rats, and mice, is a widely accepted method for assessing the toxicological and carcinogenic effects of aerosols and mists containing unknown toxic materials, including chemicals, metallic dust, nanoparticles, industrial fumes, combustion byproducts, volatile organic compounds (VOCs), particulate matter (PMs), welding fumes, and engineered nanomaterials^63–65^. These protocols provide a controlled framework to evaluate the health hazards of exposure to materials of unknown toxicity in a manner that reflects real-world conditions.

The whole-body inhalation protocol for toxicity assessment of MWFs dates back to the early 1990s. In an experiment conducted during this period, mice and guinea pigs were exposed via nebulizers to neat and used MWFs containing biocides and isothiazolines to estimate dose-response relationships and assess hypersensitivity^66^. The findings revealed that MWF exposure in both species led to lung inflammation and identified endotoxin contamination in MWFs as a significant hazard. These endotoxins exhibited dose-dependent pro-inflammatory cytokine release, elevated lung neutrophil counts, and decreased airway conductance. A whole-body inhalation study on B6C3F1 mice exposed to aerosols of unused semi-synthetic MWFs at 27 mg/m³ per day for 85 days revealed oxidative stress in the lungs of vitamin E-deficient mice. MWF exposure led to a reduction in antioxidants, resulting in increased lipid peroxidation in these mice. In contrast, no significant changes in pulmonary function, lung structure, or inflammation were observed in mice maintained on a vitamin E-enriched diet^67^. A similar whole-body exposure study on F344 rats subjected to sub-chronic inhalation of endotoxin-contaminated water-soluble MWF aerosols revealed increased white blood cell counts in peripheral blood and a significant increase in lung weights compared to rats exposed to uncontaminated MWFs. Bioanalytical findings indicated that prolonged exposure to endotoxin-contaminated MWFs induces inflammation, reduces extracellular cytokine production, and inhibits polymorphonuclear (PMN) cell migration to sites of inflammation^68^. Most studies on MWF mist exposure have primarily focused on respiratory abnormalities, inflammatory mechanisms in respiratory organs, and dermal allergies, regardless of the MWF type. In contrast, very few investigations have specifically addressed the risk of cancer, whether malignant or benign, under sub-acute, sub-chronic, or chronic exposure conditions. Consequently, the cancer risks associated with newer MWF formulations remain unclear due to a lack of comprehensive testing.

During the mid-2010s, the National Toxicology Program (NTP) of the United States Department of Health and Human Services evaluated commercial-grade MWFs such as CIMSTAR 3800 (semi-synthetic) and Trim^®^ Vx (soluble-oil) for toxicity and carcinogenicity through inhalation studies in rats and mice^69,70^. The protocols consisted of both 3-month and 2-year evaluations where the animals were exposed to MWFs at aerosol concentrations of 0, 25, 50, 100, 200, or 400 mg/m^3^ for 6 h per day, 5 days per week. Comprehensive technical reports from bioanalytical evaluations revealed that these MWFs affected various elements of the respiratory system in both rats and mice, with the primary target being the upper respiratory tract. No adverse effects of CIMSTAR 3800 exposure at various aerosol concentrations on the survival or body-weights of rodents were reported in either 3-month or 2-year studies. However, equivocal evidence of carcinogenic activity was detected in male and female Wistar Han rats, and some evidence in female B6C3F1/N mice, with increased incidences of specific tumours and non-neoplastic lesions in the respiratory system, lymph nodes, and thyroid gland in a 2-year exposure study. A 3-month inhalation study revealed significant respiratory irritation, including nasal lesions, squamous metaplasia in the larynx, and bronchiole hyperplasia, with a severity proportional to the exposure level^69^. In the case of Trim^®^ Vx, the 2-year inhalation study demonstrated clear evidence of lung carcinogenicity in mice and equivocal evidence in rats, with significant non-neoplastic lesions observed in the respiratory tract, nose, and lymph nodes across exposure groups. A 3-month exposure study revealed significant respiratory toxicity in rats and mice, including non-neoplastic lesions such as inflammation, fibrosis, and bronchiole hyperplasia in the lungs, as well as nasal and laryngeal lesions. The effects were reported to be dose-dependent, with lung fibrosis observed at exposure concentrations as low as 50 mg/m³ in rats and 100 mg/m³ in mice^70^. On the basis of the findings from the above-mentioned studies, short-term evaluations, such as 13-week studies, are clearly sufficient to identify key toxicological effects. These short-term assessments effectively capture critical respiratory outcomes—including irritation, inflammation, and early signs of tissue alteration—while minimizing the use of animals and adhering to ethical research standards.

A key contributor to the aforementioned NTP report on CIMSTAR 3800 conducted a separate characterization and comparison of CIMSTAR 3800, Trim^®^ Vx, Trim^®^ SC210 (non-chlorinated semisynthetic MWF), and Syntilo 1023 (synthetic MWF) via 13-week whole-body inhalation studies on Wistar Han rats, Fischer 344 N/Tac rats, and B6C3F1/N mice of both sexes. The animals were exposed to the MWF treatments at aerosol concentrations of 0, 25, 50, 100, 200, or 400 mg/m^3^ for 6 h/day, 5 days per week, for up to 13 weeks. Survival, body weight, histopathology, haematology and clinical chemistry, and genotoxicity were studied post-exposure to assess the toxicity of the MWFs. The results revealed respiratory tract toxicity, including pulmonary fibrosis and laryngeal lesions, with Trim VX showing the most severe effects. These studies confirm that the respiratory tract is a primary target for toxicity. Chemical components such as oils, alkanolamines, and glycol ethers, have been identified as likely contributors to toxicity^27^.

Vegetable oils, which are primarily composed of triglycerides, are generally regarded as non-toxic when aerosolized. However, studies indicate that exposure to heated vegetable oil fumes, such as those used in cooking, can lead to respiratory inflammation, with the severity increasing with exposure duration^71^. While vegetable oils alone pose minimal health risks, the addition of nanoparticles (NPs) to enhance their properties for industrial applications, such as machining, raises significant concerns. The incorporation of NPs, such as silicon dioxide (SiO_2_), alumina (Al_2_O_3_), or titanium dioxide (TiO_2_), into these oils to enhance their machinability may alter their behaviour and potentially pose risks to the respiratory system. For example, when SiO₂ nanoparticles are dispersed in triglyceride molecules such as triolein (C_57_H_104_O_6_), a commonly occurring fatty acid ester in vegetable oils, the interaction may result in the formation of surface-bound complexes or reactive intermediates. This can be represented as a *Triolein-SiO₂ interaction*: C_57_H_104_O_6_ + SiO_2_ → C_57_H_104_O_6_-SiO_2_ (a hybrid molecule or adsorption on the nanoparticle surface). Although these interactions may not directly alter the chemical structure, they can influence surface reactivity, particle size, and aerosol properties. Such changes may affect their deposition behaviour within the respiratory tract and their interaction with lung tissues upon inhalation-based exposure. This combination can generate reactive oxygen species (ROS) or interfere with biological membranes, causing oxidative stress or inflammation. Owing to their miniscule size and high surface area, NPs can penetrate deep into the respiratory system upon inhalation. A review highlighted that inhaled nanomaterials could disrupt the lung microbiome and potentially lead to respiratory diseases^72^. Similarly, a study demonstrated that inhalation of 20 nm anatase-TiO_2_ NPs induced respiratory toxicity in animal models, emphasizing the potential risks associated with inhaling NP-enhanced aerosols^73^. These findings highlight the need for comprehensive investigations into the respiratory health impacts of NP-enhanced vegetable oil aerosols, especially in occupational settings where prolonged exposure is common.

To the best of the authors’ knowledge, the toxicology and carcinogenicity of novel NPVO-MWFs have not been previously studied, particularly through whole-body inhalation protocols. The literature contains no studies on the absorption, distribution, metabolism, or excretion (ADME) of NPVO-MWFs, either in experimental animals or humans. While ADME data for some individual components of the NPVO-MWF used in this study are available in the literature, a comprehensive discussion of each component is beyond the scope of this work, which primarily aims to evaluate the mist generated by the NPVO-MWF. Moreover, no studies have been found on the respiratory toxicity specifically associated with NPVO-MWF mist exposure, and no epidemiological investigations have evaluated the carcinogenic potential of NPVO-MWFs in humans. The absence of such data underscores the need for further research to assess the potential health risks of prolonged exposure to these metalworking fluids.

**Fig. 1:**
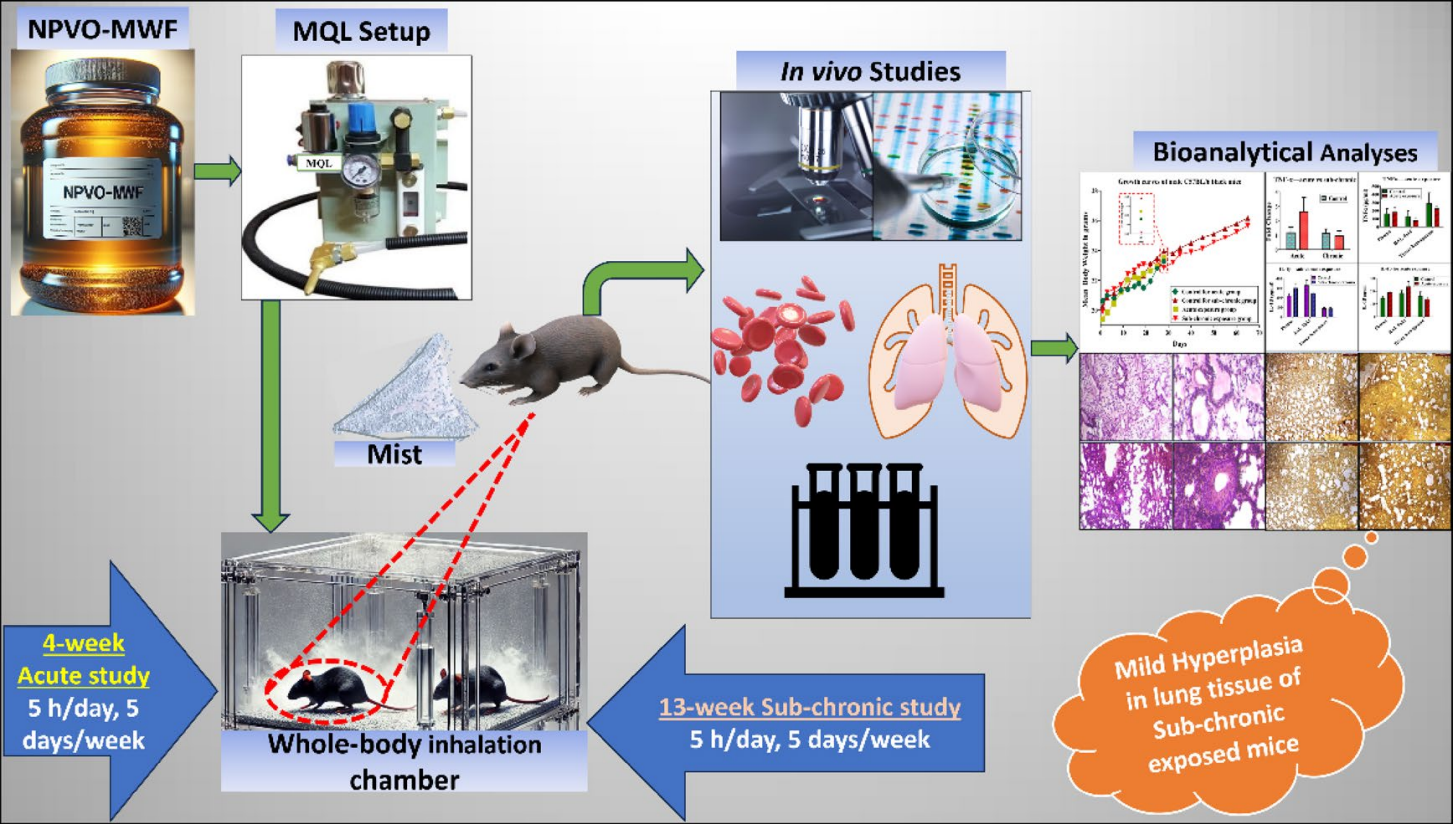
Schematic of NPVO-MWF mist generation, inhalation exposure, and biological analysis in mice in the current study.

Given this significant research gap and motivated by the findings of previous studies, the present work evaluates the sub-acute and sub-chronic effects of an in-house developed NPVO-MWF in the form of an MQL-generated mist using an animal model. Black mice were subjected to a whole-body inhalation protocol, followed by bioanalytical assessments of pulmonary and systemic toxicity, including enzyme-linked immunosorbent assay (ELISA), bronchoalveolar lavage (BAL) fluid analysis, lung tissue histology, and RNA isolation. This investigation is a critical step toward assessing the occupational health risks of novel NPVO-MWF formulations. A schematic (Fig. 1) illustrates the generation of NPVO-MWF mist, the inhalation exposure, and subsequent biological analyses in mice.

## Methods and materials

### Metalworking fluid and MQL

A sunflower oil-based nanofluid, developed in-house was used in this study. Amorphous silicon dioxide (SiO_2_) nanoparticles (CAS-No.: 7631–86 − 9) were mechanically suspended at a 1% weight to volume ratio in organic sunflower oil (CAS-No.: 8001-21-6) to prepare an NP-based vegetable-oil MWF (NPVO-MWF). The authors, in their previous studies, characterized and evaluated various other (canola and coconut oils and Al_2_O_3_ NPs) combinations of such formulations for MQL-machining of hard-to-cut materials, conducted several ecological and ecotoxicological assessments and identified the optimal formulation^61,74^. This optimal formulation has been evaluated for respiratory toxicity. The properties of the test samples used in this study are presented in Table 2. For the delivery of the developed NPVO-MWF, a state-of-the-art MQL system was used. Fresh and unused NPVO-MWF is aerosolized in the MQL-system with the help of compressed air and released in the form of a mist. To simulate a machining shop floor environment, the MQL system used in machining studies was directly employed as the input system for the inhalation chamber. The compressor air pressure was maintained at 2 kg/cm² (≈ 1.96 bar), which is consistent with the settings used in previous machining studies. The oil to compressed-air ratio was kept constant throughout all the experiments. The external mist jet spray nozzle (with a 2.3 mm diameter) features a mixing chamber where oil is drawn in and aerosolized within the nozzle before being delivered as a fine mist. The MWF flow rate was maintained at 84.6 mL/h on average.

**Table 2:** Physicochemical properties of the novel NPVO-MWF sample used in this study.

Property	Value	Apparatus	Standard
Density (g/cm^3^; 27 °C)	0.917	Digital density meter	ASTM D4052-18
Flash point (°C)—open cup	306	Abels flash point apparatus	ASTM D92
pH	8.13	pH meter with an electrode	ASTM D1293
Thermal conductivity (W/mK)	0.29	Hot disk TPS 500 analyzer	ISO 22007-2
Viscosity (cP; 27 °C)	43	Brookfield DV3T rheometer	ASTM D2983

### Inhalation chamber

**Fig. 2:**
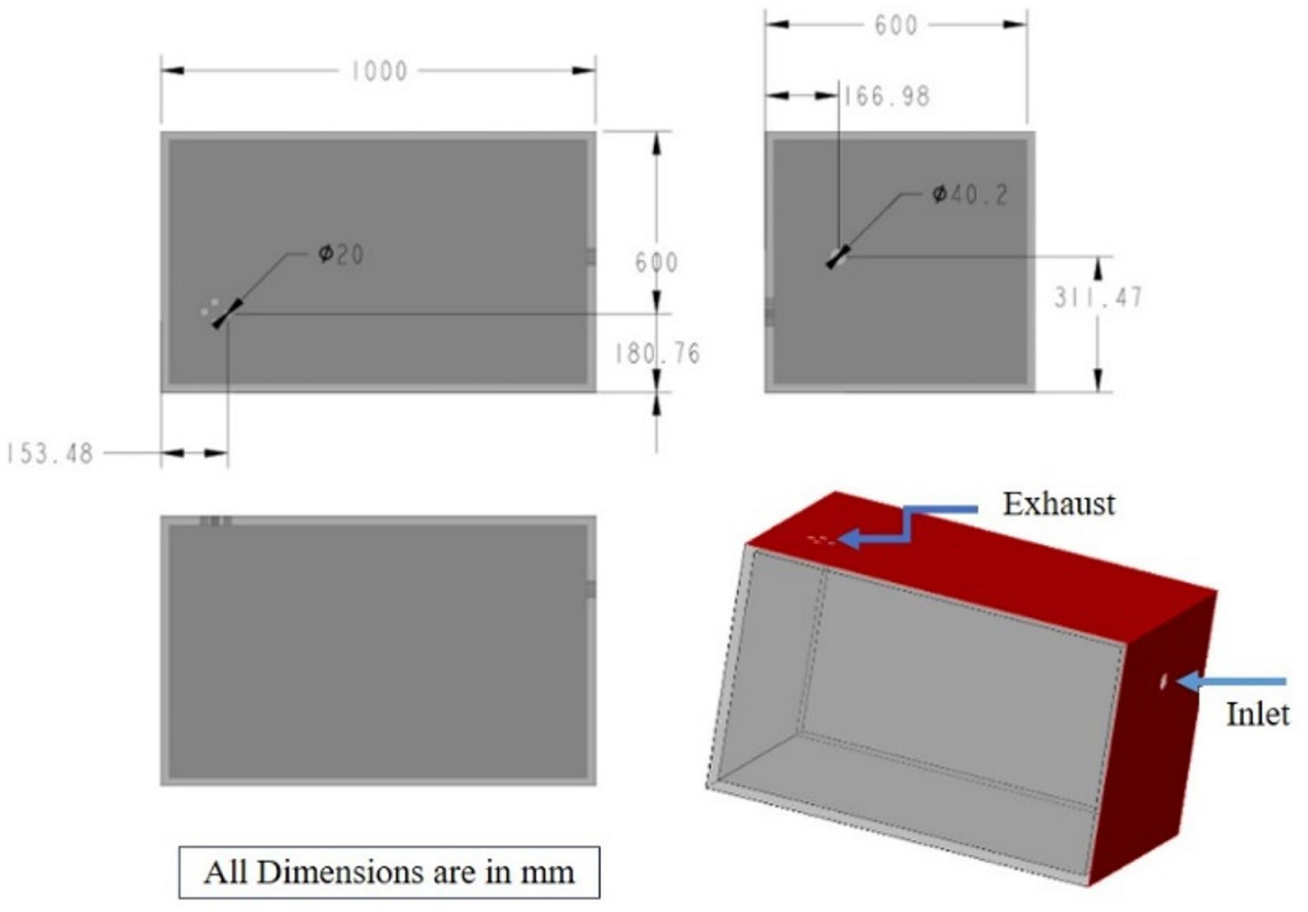
Design specifications (with all views) of the inhalation chamber used in this study.

**Fig. 3:**
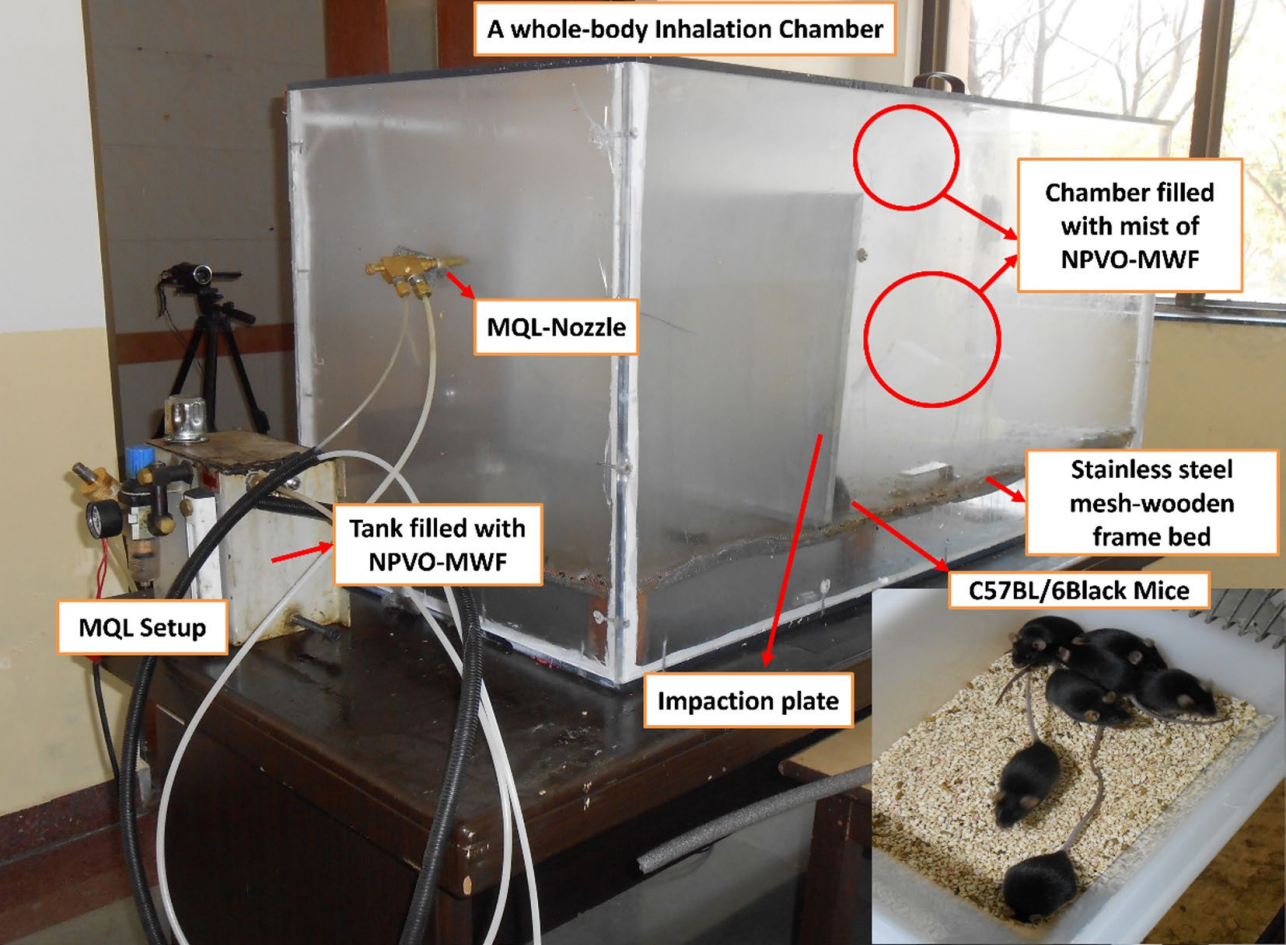
The whole-body inhalation chamber with the MQL setup and black mice used in this study.

The whole-body inhalation chamber used in this study was designed and fabricated. Figure 2 shows the design specifications of the inhalation chamber. The chamber was designed to ensure that the total volume of test animals remained below 5% of the chamber’s capacity, in compliance with OECD test guidelines. Additionally, the chamber was carefully designed to resemble the size and conditions of a typical CNC-machining room, effectively adapting the environment to suit the inhalation study requirements. The chamber was constructed from acrylic material, incorporating an inlet for the MQL nozzle. Necessary exhaust holes were provided per the design to allow the mist to flow out steadily while ensuring that the necessary mist concentration was maintained inside. A charcoal filter was fitted at the exhaust vents to filter the MWF-mist. A door on the top of the chamber facilitates easy placement and removal of the animals. The animals were placed on a stainless-steel mesh framed with wood, elevated 5 cm above the chamber floor to maintain cleanliness. To shield the animals from larger droplets, an aluminium sheet separator, otherwise called an impaction plate was positioned 40 cm from the nozzle, ensuring the animals were always placed on the opposite side. This design ensures that only fine mist and small droplets fill the chamber, as larger droplets are obstructed by the impaction plate and collected at the bottom of acrylic chamber. The inclusion of this impaction plate was based on preliminary experiments, which revealed that larger droplets caused wetting and irritation to the animals. The actual inhalation chamber with the MQL setup is shown in Fig. 3. The chamber temperature was maintained at 23 ± 2 °C. Relative humidity (RH) and CO_2_ were measured weekly, inside and outside the inhalation chamber, before and after mist generation via a portable indoor air quality probe (make: Testo). The measurements were made at different locations and the average values of RH and CO_2_ are presented in Table 3.

**Table 3:** RH and CO_2_ ranges measured at various points during inhalation experiments.

Location	RH (%)	CO_2_ (ppm)
Inside the inhalation chamber (before mist generation)	50–60	660–790
Inside the inhalation chamber (during mist generation)	60–70	1016–1518
Inside the inhalation chamber room (before mist generation)	46–55	466–580
Inside the inhalation chamber room (during mist generation)	59–68	685–754
Ambient RH outside the chamber room (25–29 °C atmospheric temperature)	41.9–59.7	439–565

### Aerosol characterization and occupational exposure limit

#### Aerosol characterization and exposure validation

The aerosolized NPVO-MWF mist was characterized using an indigenous methodology previously established by the authors, incorporating high-speed photography, laser sheet-based image capture and processing techniques, and simulation-based analysis^74^. The mean aerodynamic diameter of the droplets was determined to be 30 ± 2.19 μm. The primary objective was to characterize the naturally generated mist in machining conditions, rather than to regulate or control its particle size. The mist concentration was maintained at approximately 100 mg/m³, which is comparable to the occupational exposure levels reported for traditional MWF aerosols (NIOSH Particle Exposure Limit, PEL: 0.5 mg/m³ for MWF mist). To ensure consistency throughout the exposure duration, the chamber airflow and mist concentration were continuously monitored.

#### Occupational exposure limit (OEL)

The exposure concentration of 100 mg/m³ was determined based on a controlled whole-body inhalation chamber study that utilized a compressed air-driven MQL system to generate a stable NPVO-MWF aerosol mist. This concentration was empirically measured and validated through gravimetric sampling following the NIOSH Method 0600. The selected exposure level (~ 100 mg/m³) significantly exceeds the occupational exposure limits (OELs) recommended by NIOSH and OSHA for metalworking fluid mist (0.5 mg/m³). However, the high-intensity exposure level was designed to simulate worst-case occupational scenarios encountered in machining workshops with inadequate ventilation. While transient mist concentrations in such environments can exceed 10–50 mg/m³. Despite exceeding conventional OELs (0.5–5 mg/m³), this approach facilitates dose-response assessments, offering insights into potential sub-chronic health effects associated with prolonged NPVO-MWF mist exposure. Furthermore, the concentration emphasized in this study is consistent with previous inhalation toxicology studies on MWFs, which employed high-dose exposure models to assess respiratory toxicity in rodent models^69,70^. The absence of significant toxicological responses at this level suggests a favourable safety margin for NPVO-MWFs.

### Sub-acute and Sub-chronic studies in black mice

#### Mice

C57BL/6 black mice (male, 6 − 7 weeks old) were procured from the National Animal Resource Facility for Biomedical Research (NARFBR), Hyderabad and quarantined for 10 days upon arrival, prior to the start of the experiments. Th mice were housed in a controlled environment with a temperature of 22 ± 2 °C, 44–56% relative humidity, and a 12-hour light-dark cycle. They were provided with a standard chow diet and water *ad libitum*, and were housed in groups of five per cage with corn comb bedding. The cages were changed every alternate day. The use of animals in this study was approved by the Institutional Animal Ethical Committee under approval no. BITS-HYD/IAEC/2024-002. Animal care and use in this study complied with the guidelines established by the Committee for the Purpose of Control and Supervision of Experiments on Animals (CPCSEA), India.

#### Inhalation protocol

Four-week and 13-week whole-body inhalation studies were conducted on C57BL/6 black mice to assess the cumulative toxic effects of repeated exposure to the developed NPVO-MWF mist. The 4-week evaluation was designed as a derivative of the 13-week sub-chronic inhalation toxicity study described in OECD TG 413, providing early biological response data prior to extended exposure in line with the overall study framework.

Animals were randomly grouped as 7 (sub-acute toxicity), 7 (sub-chronic toxicity), 4 (control for sub-acute toxicity) and 4 (control for sub-chronic toxicity). A SHAM (Simulated Handling and Maintenance) control group was not included because control animals inhaling ambient air under standard housing conditions provided an appropriate baseline for evaluating NPVO-MWF mist effects. This approach was selected in the current study to minimize additional animal use, in alignment with the 3Rs principle (Replacement, Reduction, and Refinement).

*Sub-acute inhalation*.

A group of 7 mice were subjected to whole-body inhalation to the developed NPVO-MWF aerosols at a concentration of approximately100 mg/m^3^, for 5 h per day, for 5 days per week for 4 weeks. The group of 4 mice (control for sub-acute study) were left housed in the cages, except for the weekly body-weight measurements and clinical observations. The control animals were subjected to filtered air running through the Central Animal Facility.

*Sub-chronic inhalation*.

A group of 7 mice were exposed via whole-body inhalation to developed NPVO-MWF aerosols at concentration of approximately 100 mg/m^3^, 5 h per day, 5 days per week for 13 weeks. During the first 4 weeks, all 14 mice were included in the sub-acute exposure group. At the end of this period, 7 mice were randomly selected and sacrificed for bio-analytical evaluations, while the remaining 7 were retained for continued exposure until the end of the 13-week study. The group of 4 mice (control for sub-chronic toxicity) were left housed in the cages, except for the weekly body-weight measurements and clinical observations. These control animals are exposed to filtered air running through the Central Animal Facility.

#### Tissue dissection

The animals were euthanized via a high dose isoflurane anaesthesia following sub-acute and chronic exposure periods. Blood samples were collected and Bronchoalveolar Lavage (BAL) fluid was obtained by flushing the lungs with phosphate-buffered saline (PBS) for ELISA analysis. The lungs were harvested and sectioned into multiple parts for histological examination, RNA isolation, and ELISA assays.

### Histological and biochemical techniques for cellular and molecular analyses

#### ELISA

All the animals were sacrificed following sub-acute and sub-chronic inhalation exposure, and samples of blood, BAL fluid from the lungs, and tissue homogenate were collected for cytokine analysis. IL-1β (Interleukin-1 beta), IL-6 (Interleukin-6), and TNF-α (Tumor Necrosis Factor-alpha) levels were quantified via ELISA, a robust and versatile method for protein quantification in biological samples. These cytokines, which function as proteins, are key inflammatory markers, and are often referred to as pro-inflammatory cytokines. Elevated levels of IL-1β, IL-6, and TNF-α indicate the activation of inflammatory pathways, providing critical insights into the immune response triggered by exposure to foreign substances or treatments^75,76^. A persistent increase in these markers may indicate chronic inflammation, tissue damage, or potential progression to conditions such as pulmonary fibrosis or systemic inflammatory disorders^77^.

Given that the respiratory system is a primary target for MWF-mist, as established in the literature, measuring IL-1β, IL-6, and TNF-α levels in blood, BAL fluid, and tissue homogenate is crucial for assessing the inflammatory response to NPVO-MWF mist exposure. BAL fluid analysis reflects local inflammatory responses in the lungs, and is particularly relevant in inhalation exposure studies. Blood analysis offers a systemic perspective, providing an overview of the overall immune system, whereas tissue homogenate analysis focuses on localized cytokine activity in lung tissues. Together, these measurements provide comprehensive data on potential health risks associated with NPVO-MWF exposure.

In this study, commercially available kits for IL-1β (DY401-05), IL-6 (DY405-05), and TNF-α (DY410-05) from R&D Systems were utilized. High-binding 96-well plates were coated with 100 µL of capture antibody prepared in PBS and incubated overnight. After the samples were washed with PBST (0.05%), 1% BSA in PBS was added for blocking, and the samples were incubated for 1.5 h. Standards and samples prepared in 1% BSA (100 µL) were then added to the plates and incubated for 2 h. Following a wash step, 100 µL of detection antibody in 1% BSA was added, and the mixture was incubated for another 2 h. Streptavidin-HRP (diluted 1:20 in 1% BSA) was added (100 µL) and incubated for 30 min. After the final wash, 100 µL of TMB substrate was introduced and incubated in the dark for 30 min. The reaction was stopped with 2 N H_2_SO_4_, and the absorbance was recorded at 450 nm. All steps were conducted at room temperature.

#### ROS generation

Reactive Oxygen Species (ROS) are crucial for regulating normal cellular processes, but their excessive generation can lead to oxidative stress and pathological conditions^78^. Elevated levels of ROS can cause significant damage to DNA, proteins, and lipids. Additionally, ROS play a critical role in activating pro-inflammatory pathways, contributing to the development of chronic conditions such as atherosclerosis, rheumatoid arthritis, and chronic obstructive pulmonary disease (COPD)^79^. High and persistent ROS generation is implicated in tissue damage and carcinogenesis, highlighting its role in tumor development and tissue injury^80,81^.

Reactive oxygen species (ROS) levels were measured in blood, BAL fluid, and lung tissue homogenates collected from the control, sub-acute, and sub-chronic exposure groups. The samples were incubated with 10 µM 2′,7′-Dichlorofluorescin diacetate (DCF-DA) solution for 30 min. Fluorescence was measured via a Spectramax instrument at an excitation wavelength of 485 nm and an emission wavelength of 530 nm, providing quantitative data on ROS generation across the different exposure groups.

#### Real-Time polymerase chain reaction (RT-PCR)

RT-PCR is a highly effective molecular biology technique for amplifying and quantifying DNA or RNA in real time. This method has a wide range of applications in research, diagnostics, and medical sciences. It is frequently utilized to detect pathogens such as SARS-CoV-2 (COVID-19), HIV, and influenza by amplifying their genetic material^82^. In toxicology, RT-PCR plays a crucial role in analyzing changes in gene expression caused by exposure to toxic substances, including metalworking fluid (MWF) aerosols, drugs, or environmental toxins^83^.

RNA was extracted from lung tissues in this study using TRIZOL reagent (Invitrogen, USA). Following animal sacrifice, the lung tissues were promptly dissected into small pieces, and a portion was immediately preserved in TRIZOL. The samples were homogenized for one minute at medium speed using a bead homogenizer while being kept on ice to ensure effective cell lysis. Debris was removed through centrifugation at 10,000 RPM for 10 min at 4 °C. The resulting supernatant was combined with 350 µL of chloroform, incubated for 10 min with intermittent vortexing, and centrifuged at 15,000 RPM for 30 min. The aqueous phase was carefully transferred to a fresh tube, mixed with 250 µL of isopropanol, and stored at -20 °C for one hour to precipitate the RNA.

After centrifugation, the RNA pellet was washed twice with 75% ethanol, air-dried for one hour, and subsequently dissolved in nuclease-free water. The RNA concentration and purity were determined via a nanodrop spectrophotometer by measuring the 260/280 and 260/230 absorbance ratios, whereas the RNA integrity was assessed through 1% agarose gel electrophoresis. Using the Verso kit (Thermo Scientific), 1 µg of total RNA was reverse transcribed into complementary DNA (cDNA). For RT-PCR analysis, a reaction mixture containing 1 µg of cDNA, SYBR mix, and forward and reverse primers was prepared. The housekeeping gene 18 S served as an internal reference to normalize the expression of target genes. Relative gene expression levels were analyzed and compared between experimental groups using GraphPad Prism software (version 5.01). Fold changes in gene expression were calculated by comparing the target gene expression levels to the average expression levels observed in the control group.

#### Hematoxylin and Eosin (H&E)

Hematoxylin and Eosin (H&E) staining is a widely employed method in histopathology for microscopic analysis of tissue samples^84^. This method is instrumental in evaluating the effects of inhaled MWF-mist on respiratory and systemic tissues, offering insights into the toxicity and pathological alterations induced by exposure. H&E staining is essential for identifying histopathological features such as fibrosis, epithelial hyperplasia, squamous metaplasia, and necrosis, which are indicative of sub-acute or chronic toxicity caused by the MWF-mist. The histological observations obtained through H&E staining complement biomarker analyses, including cytokine quantification (e.g., IL-1β, IL-6, and TNF-α)^85^. These combined approaches help establish dose-response relationships and elucidate the pathological effects of MWF-mist exposure on mice.

In this study, lung tissues were fixed in 10% formalin and processed via graded alcohol concentrations, xylene, and paraffin wax. The tissue molds were sectioned into 5 μm slices using a microtome. For histopathological evaluation, the sections were subjected to deparaffinization, rehydration, and H&E staining. This methodology facilitated detailed visualization of cellular and tissue-level changes, contributing to a comprehensive understanding of the toxicological impact of the NPVO-MWF.

#### Sirius red staining

Sirius Red staining is a histological technique widely used to detect and quantify collagen in tissue samples^86^. Prolonged exposure to MWF mist may result in pulmonary fibrosis, characterized by excessive collagen deposition in the lungs. In inhalation studies involving mice exposed to aerosols, Sirius Red staining aids in evaluating the long-term effects of inhaled substances, assessing treatment efficacy, and understanding the pathophysiology of exposure-induced pulmonary diseases by highlighting fibrosis patterns^87^.

In the present study, lung tissues were processed for Sirius Red staining to identify fibrosis. The tissue samples were fixed in 10% formalin for 24–48 h, dehydrated sequentially using graded ethanol concentrations (70%, 80%, 95%, and 100%), and cleared with xylene prior to paraffin embedding. Thin sections of 4–5 μm thickness were cut, placed on glass slides, and deparaffinized in xylene. The sections were rehydrated through the addition of graded ethanol back to water, stained with haematoxylin for nuclear contrast, and rinsed with tap water. The samples were then stained with 0.1% Sirius Red in saturated picric acid for 1 h, followed by rinsing in acidified water (0.5% acetic acid). After dehydration of graded ethanol and clearing in xylene, the sections were mounted with a coverslip using suitable mounting medium. Under a light microscope, collagen fibers indicative of fibrosis were visualized as red, providing valuable insights into tissue remodelling.

#### Statistical analysis

All the experiments were conducted in triplicate, and the results are presented as the mean ± Standard Error of the Means (SEMs). Data analysis was performed using GraphPad Prism software (version 5.0). Statistical evaluations, including Student’s t-test, one-way ANOVA, and two-way ANOVA, were applied to assess significance, with p-values less than 0.05 deemed statistically significant. Variance across groups was found to be statistically comparable.

## Results

### Survival, body weights and clinical observations

**Fig. 4:**
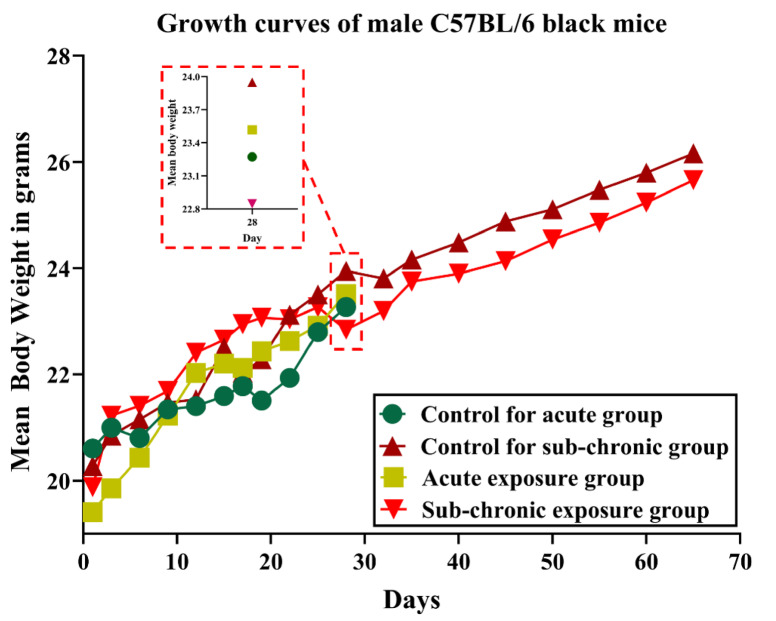
Growth curves of male C57BL/6 black mice exposed to novel NPVO-MWF via inhalation, and the control groups.

All the mice survived both the 4-week and 13-week exposure periods to the tested NPVO-MWF. By the end of their respective exposure durations, the animals in the exposed groups exhibited normal activity levels. No significant differences were observed in the mean final body weights of the exposed groups compared with the control group. Figure 4 illustrates the growth curves of all the mice across the exposure periods.

No exposure-related clinical signs, such as lethargy, were detected in either the sub-acute or sub-chronic exposure groups. The skin of the mice in the exposed groups appeared normal, with no signs of ruffled fur or changes in pigmentation. Furthermore, no visible skin abnormalities or symptoms were observed in any of the tested animals.

### Pro-inflammatory cytokines

#### IL-1β

**Fig. 5:**
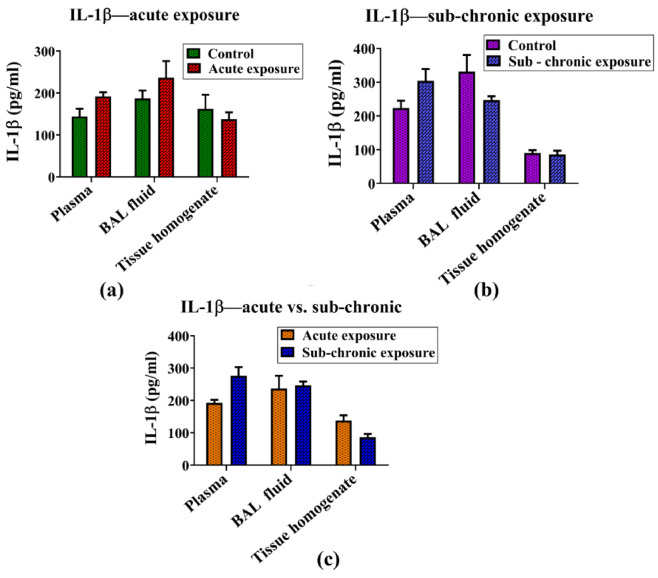
(**a**-**c**): Expression levels of the pro-inflammatory cytokine IL-1β remained unchanged following both sub-acute and sub-chronic exposure to the nano-formulation. The figure illustrates IL-1β levels measured in blood, BAL fluid, and lung tissue homogenate for (**a**) sub-acute exposure, (**b**) sub-chronic exposure, and (**c**) a comparison between sub-acute and sub-chronic exposure groups. Data were analyzed via two-way ANOVA with the Bonferroni post-hoc tests. Results are expressed as mean ± SEM, with control groups (*N* = 4) and exposed groups (*N* = 7).

The expression levels of the pro-inflammatory cytokine IL-1β remained unchanged following both sub-acute and sub-chronic exposures to the NPVO-MWF; To evaluate the potential impact of the nano-formulation on IL-1β expression, its levels were measured in blood, lung tissues, and BAL fluid via ELISA. No significant changes in IL-1β levels were observed following either sub-acute or sub-chronic exposure to the nano formulation. Furthermore, a comparative analysis of IL-1β levels between the sub-acute and sub-chronic exposure groups revealed no statistically significant differences. The results for IL-1β are shown in Fig. 5 (a-c).

#### TNF-α

**Fig. 6:**
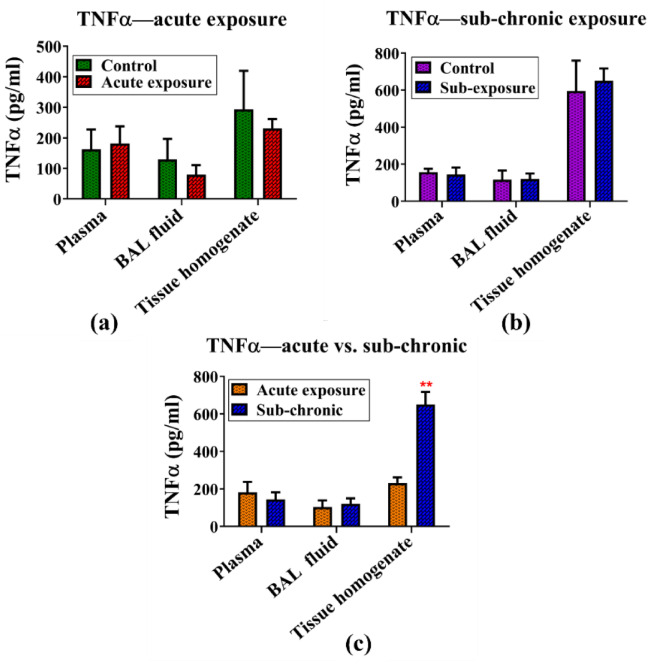
(**a**-**c**): The levels of the inflammatory cytokine TNF-α remained consistent following both sub-acute and sub-chronic exposure to the nano-formulation. TNF-α concentrations were measured in the blood, BAL fluid, and lung tissue homogenates of (**a**) sub-acutely exposed animals, (**b**) sub-chronically exposed animals, and (**c**) a comparison of sub-acute versus sub-chronic exposure. Data were analyzed via two-way ANOVA followed by the Bonferroni post-hoc test. Results are presented as mean ± SEM, with sample sizes of *N* = 4 for the control group and *N* = 7 for the exposed groups.

The expression of the inflammatory cytokine TNF-α remained unchanged following both sub-acute and sub-chronic exposure to NPVO-MWF; TNF-α levels were measured in blood, BAL fluid, and lung tissue homogenate to assess its expression following exposure to the nano-formulation. No significant changes in TNF-α levels were detected following either sub-acute or sub-chronic exposure to the nano-formulation. These findings indicate that the inflammatory response mediated by TNF-α remains unaffected under the tested exposure conditions. Additionally, a comparative analysis of TNF-α levels between the sub-acute and sub-chronic exposure groups revealed no statistically significant differences. The TNF-α results are shown in Fig. 6 (a-c).

#### IL-6

**Fig. 7:**
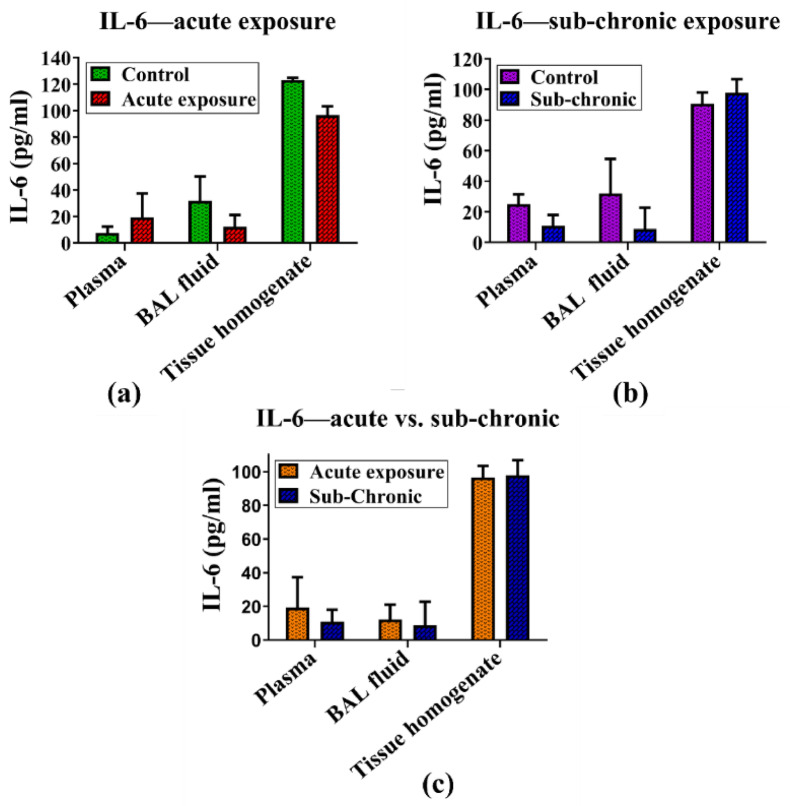
(**a**-**c**): The levels of the inflammatory cytokine IL-6 remained unchanged following both sub-acute and sub-chronic exposure to the nano-formulation. IL-6 concentrations were measured in the blood, BAL fluid, and lung tissue homogenates of (**a**) sub-acutely exposed animals, (**b**) sub-chronically exposed animals, and (**c**) a comparison of sub-acute versus sub-chronic exposure. Data were analyzed via two-way ANOVA followed by the Bonferroni post-hoc test. Results are expressed as mean ± SEM, with sample sizes of *N* = 4 for the control group and *N* = 7 for the exposed groups.

The expression of the inflammatory cytokine IL-6 remained unchanged following both sub-acute and sub-chronic exposure to the NPO-MWF; no significant changes in IL-6 levels were observed in the blood, BAL fluid, or lung tissue homogenates following either sub-acute or sub-chronic exposure to the nano-formulation. Furthermore, a comparative analysis of the IL-6 levels between the sub-acute and sub-chronic exposure groups revealed no statistically significant differences, indicating that the nano-formulation did not induce a measurable IL-6-facilitated inflammatory response under the tested conditions. The results for IL-6 are shown in Fig. 7 (a-c).

### ROS generation

**Fig. 8:**
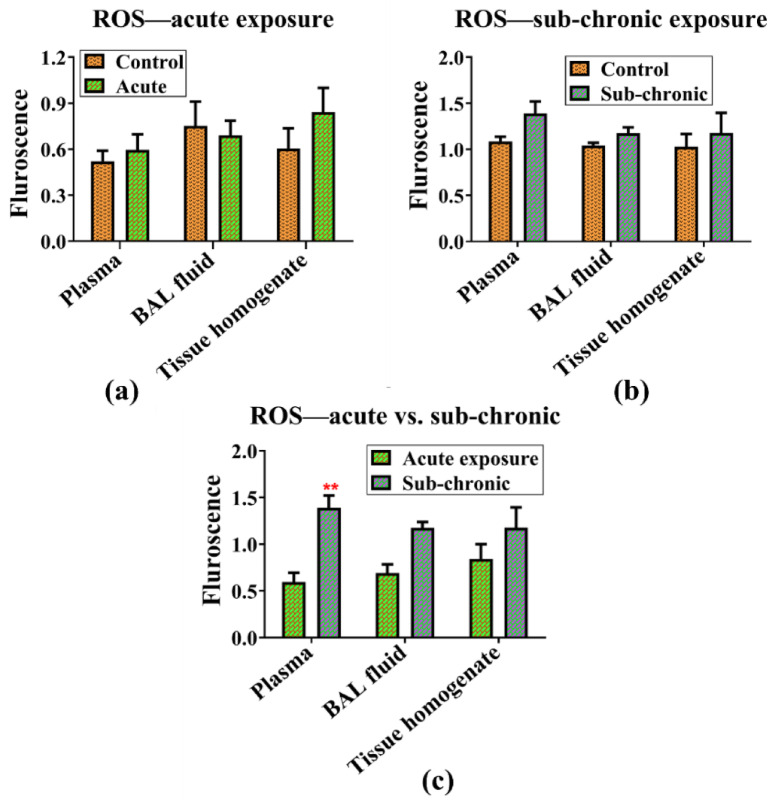
(**a**-**c**): Exposure to the nano-formulation does not induce ROS activation or apoptotic cell death. Fluorescence measurements of 2’,7’-dichlorodihydrofluorescein diacetate (DCFH-DA) staining were conducted on blood, BAL fluid, and lung tissue homogenate of (**a**) sub-acutely exposed animals, (**b**) sub-chronically exposed animals, and (**c**) a comparison of sub-acute versus sub-chronic exposure. Data were analyzed via two-way ANOVA with the Bonferroni post-hoc test, presented as mean ± SEM (*N* = 4 for control; *N* = 7 for exposed groups).

Exposure to NPVO-MWF did not induce ROS activation or apoptotic cell death in the studied animals. ROS levels were measured in blood, BAL fluid, and lung tissue homogenates following both sub-acute and sub-chronic exposure to the nano-formulation. No significant changes in the ROS levels were observed under either exposure condition. The ROS generation results are presented in Fig. 8 (a-c).

Moreover, the analysis of ROS levels between the sub-acute and sub-chronic exposure groups revealed no statistically significant differences in the BAL and tissue homogenate. A statistically significant difference was observed for blood samples as illustrated in Fig. 8-(c); however, the variation is accompanied by a substantial error bar, suggesting that the observed differences lack statistical significance and could result from variability within the data rather than a true biological effect. The observed variability in systemic ROS levels, particularly within the blood samples, suggests transient fluctuations rather than consistent oxidative stress induced by the NPVO-MWF exposure. This variability is likely attributable to inter-individual differences, circadian influences, or procedural nuances inherent in animal studies, rather than a biologically meaningful effect linked to the inhalation exposure. The absence of corresponding elevations in ROS levels in the bronchoalveolar lavage fluid and lung tissue homogenates—sites directly exposed to the inhaled mist—further supports the conclusion that the observed fluctuations do not represent a persistent or biologically relevant oxidative stress response. Such distinctions are critical for interpreting the toxicological significance of the ROS findings in the context of occupational health risks.

These findings indicate that the nano-metalworking fluid does not induce oxidative stress or contribute to ROS-mediated lung damage, even with prolonged exposure.

### RT-PCR

**Fig. 9:**
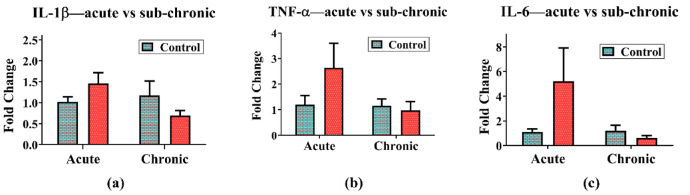
(**a**-**c**): The gene expression levels of IL-6, IL-1β, and TNF-α remained unchanged when animals were exposed to the nano-formulation under both sub-acute and sub-chronic conditions. Gene expression levels of (**a**) IL-1β (**b**) TNF-α and (**c**) IL-6 in between control and NPVO-MWF exposed animals for sub-acute and sub-chronic period. Data was analyzed by two-way ANOVA followed by Bonferroni-post comparison test, SEM (*N* = 4) in control and (*N* = 7) in exposed groups.

The gene expression levels of IL-6, IL-1β, and TNF-α remained unchanged in animals exposed to the NPVO-MWF under both sub-acute and sub-chronic conditions. The RT-PCR results are shown in Fig. 9 (a-c). The results revealed no statistically significant differences in the expression of these primary pro-inflammatory markers. These findings suggest that exposure to the nano-formulation does not result in genetic modulation of key inflammatory cytokines.

### H&E staining

Sub-acute and sub-chronic exposure to NPVO-MWF did not result in any pathological changes in the lungs, as confirmed by Hematoxylin and Eosin (H&E) staining. This staining technique is a cornerstone in lung injury assessment, providing a detailed visualization of tissue architecture. It allows for the identification of cellular damage, inflammation, and structural alterations, making it indispensable for diagnosing and evaluating lung health.

**Fig. 10:**
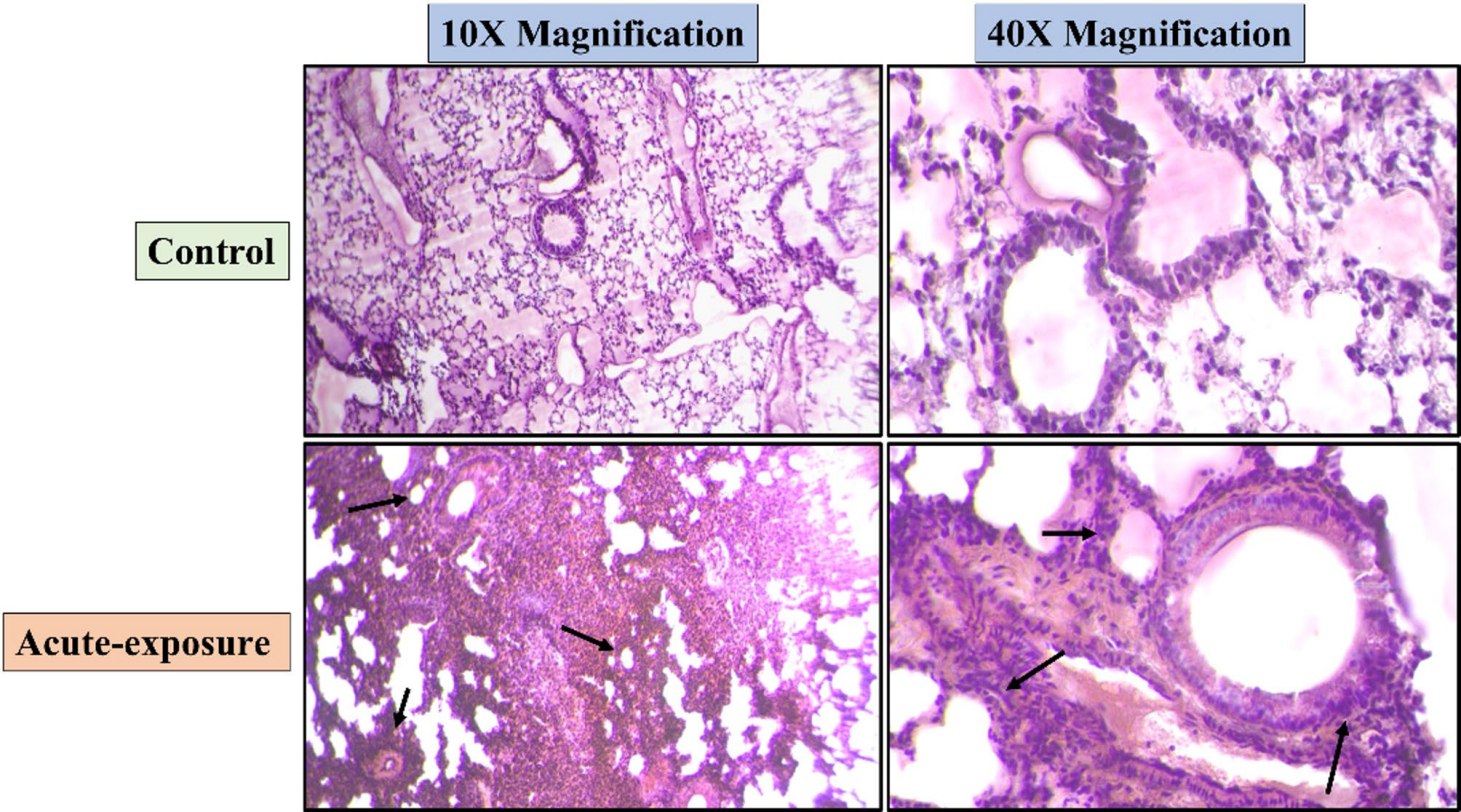
Sub-acute exposure to the nano-formulation did not result in any major observable pathological changes in lung tissues, as demonstrated by Hematoxylin and Eosin (H&E) staining. Mild accumulation of cell deposition is indicated by arrows. Representative micrographs at 10X and 40X magnifications show the histological structure of lungs following sub-acute exposure to the developed NPVO-MWF.

**Fig. 11:**
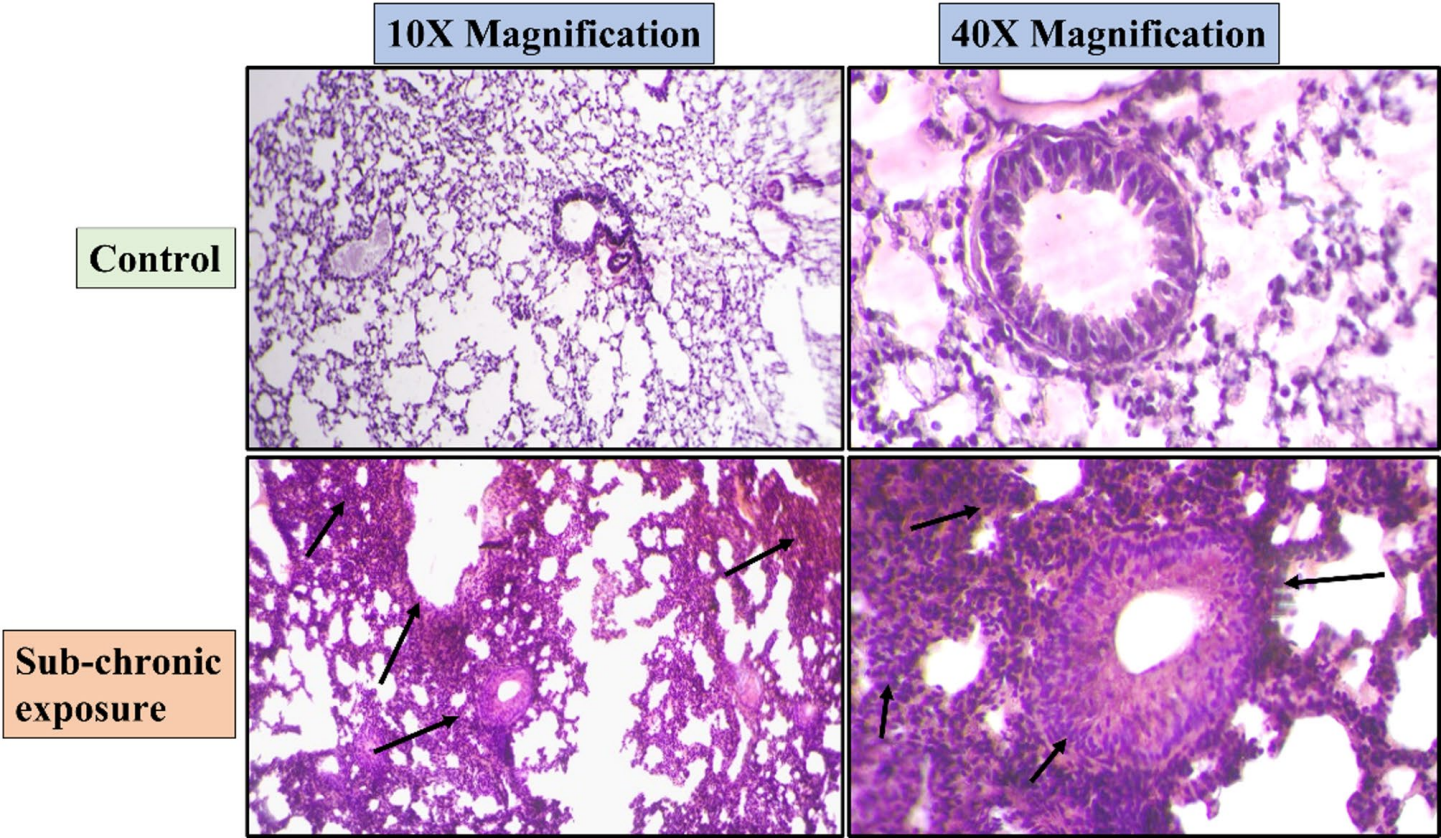
Sub-chronic exposure to the nano-formulation induced a low level of hyperplasia lung tissues denoted by arrows, as demonstrated by Hematoxylin and Eosin (H&E) staining. Representative micrographs at 10X and 40X magnifications show the histological structure of lungs following chronic exposure to the NPVO-MWF.

In this study, no significant damage to lung tissues was observed following sub-acute exposure, with Hematoxylin-Eosin-stained lung sections showing no noticeable morphological changes compared with those of the control group. However, in the sub-chronic exposure group, a low level of hyperplasia was detected, as indicated by the arrows. These findings suggest that while sub-chronic exposure to the nano-formulation has a minimal effect on lung health, mild hyperplastic changes may occur over prolonged exposure periods. Figures 10 and 11 show H&E-stained lung sections from the sub-acute and sub-chronic exposure groups respectively, with arrows highlighting any noticeable morphological changes.

### Sirius red staining

**Fig. 12:**
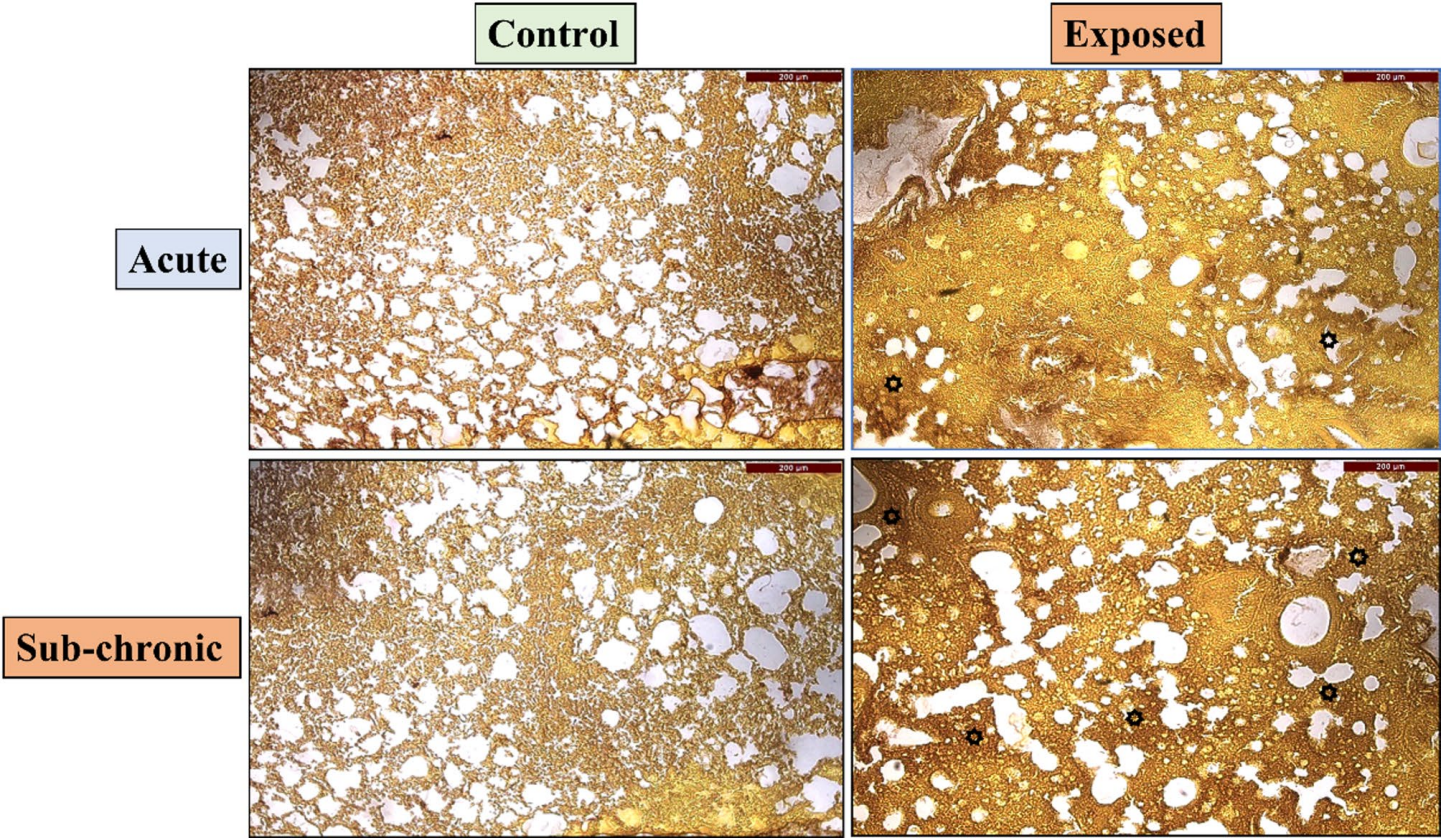
Sub-acute and sub-chronic exposure to the nano-formulation did not result in pathological changes in lung tissues, as indicated by Sirius Red staining. This staining technique was employed to assess collagen deposition in lung tissue. Representative images at 10X and 40X magnifications highlight the histological features following sub-acute and chronic exposure to the NPVO-MWF.

Sub-acute and sub-chronic exposure to the nano-formulation did not result in any pathological changes in the lungs, as evidenced by Sirius Red staining. This staining technique is highly specific to collagen fibers, making it an essential tool for detecting and quantifying collagen deposition, a hallmark of fibrotic damage in lung tissue. Figure 12 presents Sirius Red-stained lung sections from the sub-acute and sub-chronic exposure groups, with star indicating notable morphological changes.

The Sirius Red-stained sections showed minimal collagen staining, indicating that lung fibrosis was not significantly induced by the NPVO-MWF during either the sub-acute or sub-chronic exposure periods. However, in sub-chronic exposure animals, the lung sections revealed non-compact collagenous deposits in the extracellular matrix, accompanied by a slight reduction in air space. These findings suggest that the nano-formulation does not contribute significantly to fibrotic changes or compromise lung integrity over the tested exposure durations.

## Discussion

The findings of this study demonstrate that both sub-acute and sub-chronic exposure to the tested NPVO-MWFs in the form of a mist does not induce significant inflammatory or pathological responses in the lung tissue of experimental animals. This conclusion is supported by the stable levels of key pro-inflammatory cytokines, such as IL-1β, TNF-α, and IL-6, alongside the absence of oxidative stress markers and histopathological abnormalities. These results suggest that the nano-formulation, under the tested conditions, has minimal adverse effects on lung tissues.

IL-1β, a pivotal mediator in the inflammatory cascade, is known for amplifying immune responses in conditions such as sub-acute lung injury and chronic obstructive pulmonary disease (COPD)^88^. Despite its critical role in driving inflammation, the analysis of IL-1β levels in blood, lung tissues, and BAL fluid revealed no significant changes following either sub-acute or sub-chronic exposure to the mist of NPVO-MWF. This observation indicates that the MWF formulation employed in this study does not activate the inflammatory pathways commonly linked to lung injury. Similarly, the levels of TNF-α, a cytokine closely associated with lung inflammation and fibrosis, were consistent across biological compartments, including blood, BAL fluid, and tissue homogenate, regardless of the exposure duration. The stable levels of TNF-α reinforce the hypothesis that NPVO-MWF mist does not provoke a pro-inflammatory response, which is often a precursor to more severe lung damage, such as fibrosis^89^. IL-6, another cytokine implicated in lung diseases such as sub-acute respiratory distress syndrome (ARDS), plays a role in Th17 cell differentiation and the sub-acute phase response, which can worsen respiratory conditions. However, no significant changes in IL-6 levels were observed under sub-acute or sub-chronic exposure to the NPVO-MWF mist. The absence of significant alterations in IL-6 levels after both sub-acute and sub-chronic exposure suggests that the nano-formulation does not induce an inflammatory environment conducive to lung injury or disease progression^90^.

Additionally, the potential for oxidative stress, a major contributor to lung injury due to the damaging effects of ROS, was investigated. The results revealed no significant increase in the ROS levels in either the blood, BAL fluid or tissue homogenate indicating that NPVO-MWF mist does not induce oxidative damage in lung tissues^91^.

At the molecular level, gene expression analyses of IL-6, IL-1β, and TNF-α through RT-PCR revealed no significant alterations following sub-acute or sub-chronic exposure to the nano-formulation. The observed absence of pro-inflammatory genetic modulation suggests that NPVO-MWF does not induce significant immune responses. This finding highlights its potential as a safe option for machining operations. Even under conditions of prolonged exposure to mist, NPVO-MWF demonstrates promising safety characteristics, making it a viable alternative for use in industrial settings. Its minimal immunogenic profile supports its suitability for environments where operator health and safety are critical. However, further research is necessary to comprehensively evaluate its long-term immunological impacts.

Histopathological analysis via H&E staining confirmed the presence of mild hyperplasia following sub-chronic exposure. Additionally, Sirius Red staining, which is used to detect collagen deposition as a marker of fibrosis, revealed minimal non-compact collagen accumulation and slight reductions in the airspace within the lungs of sub-chronic exposure animals. The results, illustrated in Figs. 5 and 7, revealed variability, which was partially attributed to increased margins of error. Notably, abnormalities were notably observed at the sub-chronic exposure level. Despite these findings, the cumulative bioanalytical assessment suggested that the nano-formulation is relatively safe under the tested conditions, particularly in terms of respiratory impact.

In this study, bioanalytical tests were performed on blood, BAL fluid, and lung tissue homogenate, given that the respiratory system—particularly the lungs—is widely recognized as the primary target of metalworking fluid (MWF) exposure. This study provides valuable insights into the sub-acute and sub-chronic effects of NPVO-MWF mist exposure. However, it was conducted with a limited sample size, which may limit the generalizability of the findings. Under the experimental conditions, no significant changes in lung morphology, cytokine levels, or oxidative stress markers were observed. Nonetheless, mild hyperplasia and minor indications of fibrosis were noted after sub-chronic exposure. Although not statistically significant, the slight increases in IL-1β and TNF-α gene expression in lung tissues may indicate early biological responses that warrant further evaluation under prolonged exposure. Similarly, the mild hyperplastic changes observed in lung tissues could represent early signs of tissue remodelling. The question of whether these effects are transient or progressive requires longer-term chronic exposure studies to fully address, including positive control comparisons to conventional MWFs for more comprehensive risk assessment.

Future investigations involving larger animal cohorts and extended exposure durations are recommended to establish a more comprehensive safety profile and confirm these observations. Importantly, no clear, equivocal, or substantial evidence of pulmonary toxicity or carcinogenicity was observed in the 4-week sub-acute and 13-week sub-chronic exposure of male C57BL/6 black mice to the novel NPVO-MWF mist.

Although acute and sub-chronic toxicity assessments indicate minimal adverse effects of NPVO-MWF exposure, further studies are needed to understand the toxicokinetic (ADME) of NPVO-MWFs. Notably, this 13-week sub-chronic exposure study—aligned with NTP protocols—revealed no carcinogenic findings based on ROS, cytokine, and histopathological analyses. Although the present study demonstrates minimal respiratory toxicity in the tested NPVO-MWF formulation, it is recognized that 13-week inhalation studies, while standard for sub-chronic hazard identification, may not fully characterize the potential for progressive or chronic outcomes. Therefore, chronic exposure studies (e.g., 2-year inhalation studies) and assessments in other species are recommended to build on the findings of this study and develop a comprehensive understanding of the formulation’s long-term safety and occupational health implications. Chronic (2-year) evaluations remain essential for definitive carcinogenicity assessment. Given that nanoparticles exhibit unique translocation patterns, further investigations should focus on nanoparticle distribution beyond the lungs. This includes systemic circulation, liver, and renal clearance pathways. Future research incorporating biodistribution studies using radiolabelled NPs or mass spectrometry-based bioanalytical approaches would provide valuable insights into the long-term occupational exposure risks of NPVO-MWFs.

## Conclusions

In conclusion, the comprehensive evaluation of pro-inflammatory cytokines, oxidative stress markers, and histopathological changes demonstrated that the tested NPVO-MWF formulation did not induce significant lung injury under the conditions studied. The findings highlight the viability of this nano-formulation as a sustainable and safer option for metalworking fluid mist applications, supporting occupational health and the environment. However, the assessment of carcinogenic activity remains incomplete. Current studies are constrained by qualitative and quantitative limitations, leaving the evidence inconclusive regarding potential carcinogenic effects. These findings underscore the need for further research to establish a robust understanding of the long-term safety and sustainability of nano-formulation.

While the findings suggest minimal respiratory toxicity of the NPVO-MWF mist under the tested conditions, it is important to note that the current data alone are not sufficient to fully conclude on its occupational safety. Additional studies are necessary to confirm these preliminary observations. These include long-term inhalation exposures, larger animal sample sizes, and direct comparisons with conventional MWFs to comprehensively evaluate the formulation’s occupational health and environmental sustainability profile. Future investigations should include chronic exposure studies, such as 2-year inhalation experiments, and expand to different species, such as rats, to provide a more comprehensive safety profile. These investigations are crucial for validating the safety of nano-formulations, enhancing their market readiness, and reinforcing their potential as a sustainable solution for machining environments. These efforts will facilitate its adoption in industrial applications, ensuring both environmental responsibility and operator safety.

## Data Availability

All data generated or analyzed during this study are included in this published article. Any additional information related to this work is available from the corresponding author upon reasonable request.

## Declarations

### ARRIVE guidelines compliance

This study was conducted and reported in accordance with the ARRIVE 2.0 (Animal Research: Reporting of In Vivo Experiments) guidelines to ensure transparent and comprehensive reporting of in vivo animal research. All aspects of study design, including randomization, blinding, outcome measures, and statistical analysis, were addressed to enhance reproducibility and uphold animal welfare standards.
